# *PARP1* Gene Knockout Suppresses Expression of DNA Base Excision Repair Genes

**DOI:** 10.1134/S1607672922700028

**Published:** 2023-01-18

**Authors:** A. L. Zakharenko, A. A. Malakhova, N. S. Dyrkheeva, L. S. Okorokova, S. P. Medvedev, S. M. Zakian, M. R. Kabilov, A. A. Tupikin, O. I. Lavrik

**Affiliations:** 1 Institute of Chemical Biology and Fundamental Medicine, Siberian Branch of the Russian Academy of Sciences, Novosibirsk, Russia; 2 Federal Research Centre Institute of Cytology and Genetics, Siberian Branch of the Russian Academy of Sciences, Novosibirsk, Russia; 3 Meshalkin National Medical Research Center of the Ministry of Health of the Russian Federation, Novosibirsk, Russia; 4 AcademGene LLC, Novosibirsk, Russia

**Keywords:** PARP1, *PARP1* knockout, DEG, DNA Base Excision Repair

## Abstract

The effect of *PARP1* knockout in HEK293 cells on the gene expression of DNA base excision repair (BER) proteins was studied. It was shown that the expression of all differentially expressed genes (DEGs) of BER was reduced by knockout. The expression of the DNA glycosylase gene *NEIL1*, which is considered to be one of the common “hubs” for binding BER proteins, has changed the most. The expression of genes of auxiliary subunits of DNA polymerases δ and ε is also significantly reduced. The *PARP1* gene knockout cell line obtained is an adequate cell model for studying the activity of the BER process in the absence of PARP1 and testing drugs aimed at inhibiting repair processes. It has been found for the first time that knockout of the *PARP1* gene results in a significant change in the level of expression of proteins responsible for ribosome biogenesis and the functioning of the proteasome.

Poly[ADP-ribose]polymerase 1 (PARP1) is the most widely abundant member of the extensive PARP family, which is present in the nucleus and, to a lesser extent, in the cytosol of the cell [1]. PARP1 catalyzes the synthesis of the poly(ADP-ribose) (PAR) polymer using NAD^+^ as a substrate. PAR is a branched polymer up to 200 units long, which is transferred to target proteins, covalently attached to them as a post-translational modification, including to PARP1 itself (autoPARylation), as well as to DNA and RNA [2–4]. PARylation is an immediate cell response to DNA damage that plays an important role in maintaining genome integrity and cell survival [2, 5]. PARP1 controls many processes in the cell through post-translational modification of target proteins, direct protein-protein interactions, as a transcription factor, using the free PAR polymer, and also through participation in NAD^+^ metabolism [2, 6]. In addition, PARP1 is involved in chromatin decondensation processes [7].

PARP1 is a key regulator of the base excision repair (BER) process, which is responsible for the elimination of DNA damage that does not violate DNA structure (damage or loss of bases, single-strand breaks) [8, 9]. BER was found in all organisms. BER repairs DNA damage in both the nuclear and mitochondrial genomes. BER enzymes form temporary complexes of different compositions depending on the type of damage that occurs during this dynamic process, which occurs with the transfer of damaged DNA from one complex to another [9]. Access to DNA damage in the chromatin structure and assembly of BER protein complexes requires an additional level of regulation and coordination. This role is performed by post-translational modifications, including PARylation. Briefly, BER is primarily initiated by one of eleven damage-selective DNA glycosylases [10, 11]. In addition, BER can also include several subpathways that are realized depending on the type of damage, including single-strand break repair (SSBR), where the DNA backbone has already been cleaved, and nucleotide incision repair (NIR), a minor subpathway where APE1 initiates DNA-glycosylase independent repair of oxidized bases [12, 13]. All these pathways operate concurrently with PARP1 activation (and PARP2 contribution), leading to covalent modification (PARylation) of PAR-accepting proteins and to PAR-dependent recruitment of the main repair proteins to the DNA damage site [8, 9].

*PARP1* knockout cells and mice survive [14]. This is probably due to the presence of the PARP2 enzyme in the cells [2]. Mice with *PARP1* and *PARP2* genes double knockout were not viable at an early stage of embryogenesis [15]. However, it has been shown that DNA damage repair by the BER system occurs less efficiently in cells and organisms with an inactivated *PARP1* gene, PARP1 mutations and inhibition of this enzyme cause a high degree of genomic instability in cells [2]. Transcriptomic analysis of *Parp1*(-/-) mice showed that they were protected from colitis, but this protection was associated with transcriptional reprogramming in the colon [16]. Inhibition of PARP activity renders mice susceptible to carcinogenic agents in various tumor models, but knockout mice were not prone to developing tumors [17]. Inhibition of PARP1 by small molecule NAD^+^ analogs leads to the suppression of the DNA nucleotide excision repair (NER) and base excision repair (BER) pathways and also stabilizes the PARP1-DNA complex, which leads to the accumulation of double-strand breaks [18].

In this work, we studied the effect of *PARP1* gene knockout on the expression of BER genes in the HEK293 cell line obtained using CRISPR/Cas9 technology. Cells with deletion of coding exons 3–5 were generated as in [19]. PCR analysis showed a homozygous deletion in the target gene [19]. To obtain the total RNA wild-type HEK293 cells (WT) and *PARP1* gene knockout cells (PARP1-KO) were grown in a six-well plate till the formation of a 30–50% layer (1–2 million cells per well). For each sample, there were four replicates. The cells were grown in DMEM/F12 medium (Thermo Fisher Scientific, Waltham, MA, USA), with 1×GlutaMAX (Thermo Fisher Scientific, Waltham, MA, USA), 100 IU/mL penicillin, and 100 µg/mL streptomycin (Thermo Fisher Scientific, Waltham, MA, USA), and in the presence of 10% fetal bovine serum (Biolot, Saint-Petersburg, Russia) in 5% CO_2_ atmosphere. Total RNA was isolated with the Trizol (Invitrogen, Carlsbad, CA, USA) reagent according to the manufacturer’s protocol. The quantification and quality analysis of RNA was performed on a Bioanalyzer 2100 (Agilent, Santa Clara, California, USA).

To determine the effect of knockout on gene expression, after mRNA isolation with NEBNext Poly(A) mRNA Magnetic Isolation Module (NEB, USA) the cDNA library was obtained using the MGIEasy RNA Directional Library Prep Set (MGI Tech Co., Ltd., China) and was sequenced with a coverage of 30 million paired readings with a reading length of 100 nucleotides on MGIseq 2000, BGI. The resulting reads were aligned to the human genome (hg38, ensembl v38.93) using STAR-2.7.8. Quantification of reads was also performed using STAR, option–quantModeGeneCounts. The resulting expression matrix was imported into R and analyzed using the DEseq2 package. Low-expression genes (sum of reads across all samples less than 10) were removed from the expression matrix. VST normalization was used for principal component analysis. Differentially expressed genes (DEG) were determined using the Wald test. Genes were considered differentially expressed when the p-value was 0.01 (after adjusting for multiple comparisons). Signaling pathway enrichment analysis was performed using the fgsea package (p-value 0.001) with the KEGG database.

More than 4,000 differentially expressed genes (DEGs) were found in PARP1-KO cells compared to wild-type cells (the expression level of 2253 genes was up-regulated and 2079 genes were down-regulated). Analysis of signaling pathway enrichment was performed using the fgsea package in the KEGG database. The expression of genes involved in various pathways has changed: ribosome biogenesis, antigen processing and presentation, metabolism of xenobiotics, and others, which confirms the important role of PARP1 in the functioning of different processes and the maintenance of cellular homeostasis (Fig. 1). The goal of our long-term studies was to establish the role of PARP1/2 in the regulation of the BER process, so we were interested in the effect of PARP1 knockout on this process [9]. The results of the analysis showed that in PARP1-KO cells, the expression level of ten genes encoding BER enzymes and protein factors was changed (Table 1).

**Fig. 1.**
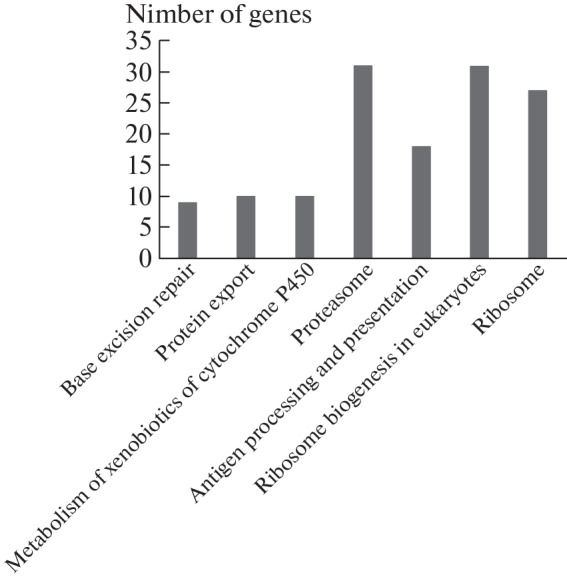
Signaling pathways that were the most strongly altered by *PARP1* knockout in HEK293 cells according to the KEGG database.

**Table 1.** Differentially expressed genes involved in the process of DNA base excision repair in PARP1-KO cells

Name	Transcript.ID	log2FoldChange	p-value	p-adj
PARP1	ENSG00000143799	–4.382	0.0e+00	0.0e+00
NEIL1	ENSG00000140398	–2.04	6.1e-17	5.3e-15
POLE4	ENSG00000115350	–1.559	2.8e-10	7.4e-09
POLD2	ENSG00000106628	–0.736	6.0e-09	1.2e-07
SMUG1	ENSG00000123415	–0.702	1.1e-05	9.9e-05
FEN1	ENSG00000168496	–0.433	2.0e-04	0.001
POLB	ENSG00000070501	–0.566	4.0e-04	0.002
MPG	ENSG00000103152	–0.896	5.9e-04	0.003
NEIL3	ENSG00000109674	–0.535	0.004	0.017
POLD4	ENSG00000175482	–1.766	0.012	0.039

As can be seen from Table 1, the level of expression of all DEGs decreased in PARP1-KO cells, to varying degrees, compared with wild-type HEK293 cells. The expression of NEIL1 DNA glycosylase, the fourth subunit of DNA polymerase ε POLE4, and the fourth subunit of DNA polymerase δ POLD4 decreased most significantly.

We built a network of protein-protein interactions (PPI) for DEGs (Table 1) using the Cytoscape 3.7.2 tool (Institute of Systems Biology, USA). The PPI network was built with a minimum required interaction score 0.7. We found that FEN1 and NEIL1 were the main “hubs” of this interaction (Fig. 2). Four of the DEGs were genes encoding DNA glycosylases (NEIL1, SMUG1, MPG, NEIL3), and four were genes encoding DNA polymerases (POLE4, POLD2, POLD4, POLB).

**Fig. 2.**
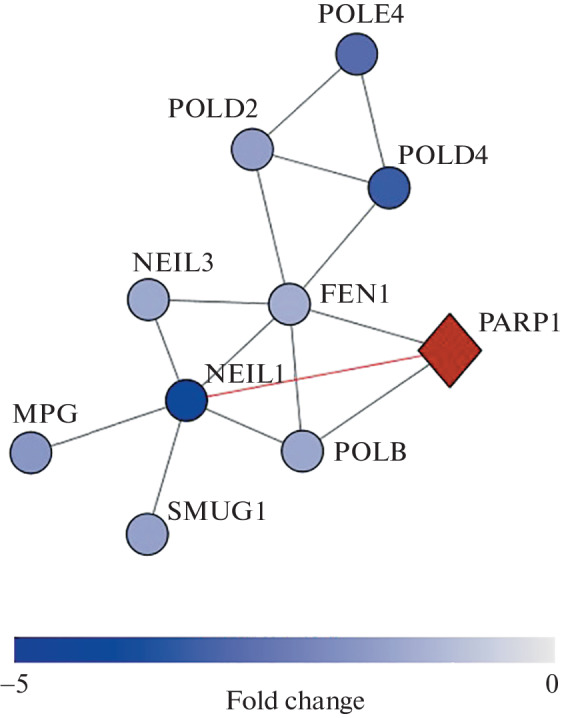
Network of protein-protein interactions of PARP1-dependent genes (blue circles) obtained using the STRING tool (minimum required interaction score 0.7). Red diamond – PARP1. The association between PARP1 and NEIL1 is highlighted in red [20]. The color intensity of nodes depends on the fold change in expression in PARP1 knockout cells compared to wild type cells. Rendered with Cytoscape v3.7.2.

NEIL1 glycosylase (Nei Like DNA Glycosylase 1) initiates BER by removing damaged nitrogenous bases, mainly oxidized pyrimidines [21]. Previously, it was shown that NEIL1 directly interacts with PARP1 by binding its C-terminal domain to the BRCT domain of PARP1, and this interaction leads to the suppression of NEIL1 activity, regardless of PARP1 activation and PAR synthesis [20]. The authors of this work [Coordination of DNA repair by NEIL1 and PARP-1] also studied the effect of free PAR, DNA-binding, catalytic, and BRCT domains of PARP1 on NEIL1 activity and concluded that protein-protein interactions are responsible for this effect. PARP1 binds to amino acid residues 289-390 localized in the C-terminal domain of NEIL1 [20]. This region serves as a “hub” for other proteins involved in subsequent BER steps, including FEN1, DNA polymerase β (Polβ), LigIIIα, and PCNA [22–24]. Our data support the idea proposed by the authors of the work [20] that NEIL1 acts as a common interface for BER protein binding.

SMUG1 (Single-Strand-Selective Monofunctional Uracil-DNA Glycosylase 1) is a DNA glycosylase that removes uracil from single- and double-stranded DNA [25, 26]. MPG (N-Methylpurine DNA Glycosylase) is a DNA glycosylase that recognizes in DNA and removes alkylated and deaminated purines [27]. NEIL3 is a DNA glycosylase from the Fpg/Nei family with lyase activity. There is no data yet on the interaction of these BER-initiating glycosylases with PARP1.

DNA polymerases δ and ε are replicative polymerases, DNA polymerase δ is mainly responsible for the synthesis of the lagging strand, and DNA polymerase ε is responsible for the synthesis of the leading strand [28]. Both of these enzymes participate in the long-patch BER, when DNA synthesis is carried out with strand displacement [29, 30], but in the case of blocking damage (e.g., apurine-apyrimidine site in the template), their ability to do synthesis through these damages is limited. In the case of blocking of processive replicative DNA polymerases, they can be replaced by repair DNA polymerases [31]. The auxiliary subunit of DNA polymerase ε POLE4 is a histone chaperone, which, in combination with the POLE3 subunit, participates in the assembly of the nucleosome due to its ability to bind selectively to histones H3-H4 [32, 33]. Subunits of DNA polymerase δ POLD2 and POLD4 are involved in the formation of the replication fork and stabilization of the replication complex [34].

It is interesting that PAR was found in normal cells in S-phase at DNA replication loci, without increased levels of DNA damage. The authors of [35] showed that PARP1 is a sensor of unligated Okazaki fragments during DNA replication and promotes their ligation, while inhibition of FEN1 (flap endonuclease 1) contributes to the accumulation of PAR.

FEN1 processes the 5' ends of Okazaki fragments in replicative lagging DNA synthesis and removes 5' overhangs during DNA repair. FEN1 inhibits strand displacement DNA synthesis on a substrate containing an AP site in a template catalyzed by DNA polymerase δ and is required for strand displacement synthesis on the same substrate catalyzed by DNA polymerase β [36]. In the same work, DNA polymerase β was shown to interact physically with FEN1. The less efficient DNA repair seen in PARP1-deficient cell extracts was associated with reduced cellular expression of several factors required for long patch BER, including FEN1 and DNA ligase I [37]. In the work [38] it has been shown that DNA translesion synthesis through damage (AP site) requires the replacement of replicative DNA polymerase ε with repair DNA polymerases β and λ. Thus, in the course of long-patch BER, the functions of various DNA polymerases can be interdependent and interchangeable. DNA polymerase β catalyzes synthesis through the damage in the presence of DNA polymerase ε, but requires short gaps in front of the AP site. Direct interaction of DNA polymerase β with PARP1 has also been shown [39]. It is likely that PARP1 is involved in switching BER from one polymerase to another, however, the mechanism of the effect of PARP1 on BER activity remains unclear.

In conclusion, it should be said, that it is interesting to study the role of PARP1 in the BER system both in vitro and in vivo. In this work, we shown that the level of genes expression of several proteins participating in BER in HEK293 cells with a knockout of the *PARP1* gene was changed, it can influence the regulation of the activity of this process. The *PARP1* knockout cell line we obtained is an adequate cell model for studying BER processes and testing drugs aimed at inhibiting repair processes. It was shown that the level of expression of genes of several key proteins participating in BER was reduced in the cells of the resulting cell line. Also of great interest is a significant change in the level of expression of proteins responsible for the biogenesis of ribosomes and the functioning of the proteasome, discovered for the first time during the knockout of the *PARP1* gene.
